# Sporadic Diffuse Palmoplantar Keratoderma in a Pediatric Patient With Early Onset: A Case Report

**DOI:** 10.7759/cureus.100318

**Published:** 2025-12-29

**Authors:** Lathika Premkumar, Sai Preethi P, Adikrishnan Swaminathan, Murugan Sundaram

**Affiliations:** 1 Dermatology, Venereology and Leprosy, Sri Ramachandra Institute of Higher Education and Research, Chennai, IND; 2 Dermatology, Sri Ramachandra Institute of Higher Education and Research, Chennai, IND

**Keywords:** acitretin, palmar hyperkeratosis, palmoplantar keratoderma, retinoid therapy, sporadic palmoplantar keratoderma

## Abstract

Palmoplantar keratoderma (PPK) encompasses a heterogeneous group of disorders characterized by hyperkeratosis of palms and soles. Sporadic cases with early childhood onset but no family history represent a diagnostic challenge. In this report, we present a case of an eight-year-old male child who presented with progressive thickening of palmoplantar skin since he was two years old, which progressed with painful fissures, ultimately resulting in restriction of mobility. The clinical examination revealed diffuse, yellow-orange hyperkeratotic plaques with well-demarcated margins and deep fissures over pressure points. On a series of follow-up visits, a complete clinical evaluation coupled with unremarkable laboratory findings ruled out syndromic associations. The diagnosis of sporadic diffuse non-transgradient PPK was made based on the inference of clinical presentation and exclusion of differential diagnoses. Treatment with low-dose acitretin (10 mg twice weekly) combined with topical keratolytic agents over three months resulted in significant clinical improvement, with marked reduction in hyperkeratosis and resolution of painful fissuring. This case highlights the importance of distinguishing isolated PPK from syndromic forms in pediatric patients. Systemic retinoid therapy proved effective in this case of early-onset sporadic PPK, significantly improving the patient's quality of life and mobility.

## Introduction

Palmoplantar keratoderma (PPK) is a heterogeneous group of disorders characterized by the presence of marked hyperkeratosis of the epidermis on the palms and soles. Unna provided a comprehensive classification of PPK in 1883 [1], and it has an estimated prevalence of 1.17/100,000 population, with variations across geographic regions and ethnic groups [2]. The condition may present as an inherited trait with several patterns of inheritance or as an acquired disorder secondary to various systemic conditions. PPK is classified into three major categories: diffuse (uniform palmoplantar thickening), focal (localized plaques), and punctate (small discrete keratotic papules).

PPK features hyperkeratotic thickening of palms and soles, classified clinically as diffuse (uniform involvement), focal (pressure points), punctate (small bumps), or striate; forms may show transgradient extension (beyond palms/soles) or remain non-transgradient (confined to sites). Inherited types arise from keratin gene mutations (e.g., KRT1, KRT9 for diffuse; AAGAB for punctate); acquired forms link to malignancies, infections, or drugs; and syndromic variants associate with Vohwinkel (deafness), Mal de Meleda (consanguinity), or pachydermoperiostosis (periostosis). These patterns direct genetic testing or malignancy screening for precise diagnosis.​

PPKs represent disorders of epidermal homeostasis, which specifically involve abnormalities in keratinocyte proliferation, differentiation, or adhesion. The hereditary forms of the syndrome typically involve mutations in genes encoding structural proteins (keratins, desmosomal components), signaling pathways (Notch), or proteins that are involved in lipid metabolism. These mutations disrupt epidermal barrier function, resulting in the characteristic hyperkeratosis. Sporadic cases may arise from de novo mutations or may represent acquired forms with distinct pathophysiological mechanisms [3].

PPK causes significant morbidity through pain, functional impairment, and reduced quality of life. The condition presents a diagnostic challenge due to its heterogeneity and potential association with multisystem disorders. Early and accurate diagnosis is crucial for implementing appropriate treatment strategies and screening for syndromic associations that may require additional interventions.

This case report documents an atypical presentation of PPK with onset in early childhood (two years of age) with no family history, representing a sporadic form. The case is notable for its favorable response to systemic retinoid therapy, which is not universally observed in PPK. Documentation of such cases contributes to the understanding of treatment response predictors and the natural history of sporadic PPK in pediatric populations.

## Case presentation

An eight-year-old male student was brought to the Outpatient Department with chief complaints of thickening of skin on his palms and soles for the past six years. The condition had begun at the age of two years as a mild thickening of the skin and had gradually progressed, resulting in fissures, especially on the weight-bearing areas of the feet. The child also had difficulty walking long distances or running due to pain emanating from these fissures. There was no history of blistering, erythema, or pruritus in the affected areas.

The patient had reached normal developmental milestones and was doing well in school. There was no history of fever, hearing impairment, visual disturbances, or other systemic symptoms that might suggest a syndromic form of PPK. Extreme callus formation and a similar skin condition were not present in any family members. The patient had no history of long-term infections or severe environmental exposures that could have contributed to the acquired keratoderma. Prior to presentation, the child had no noteworthy medical history and was not taking any long-term medications. His vital signs were normal.

**Figure 1:**
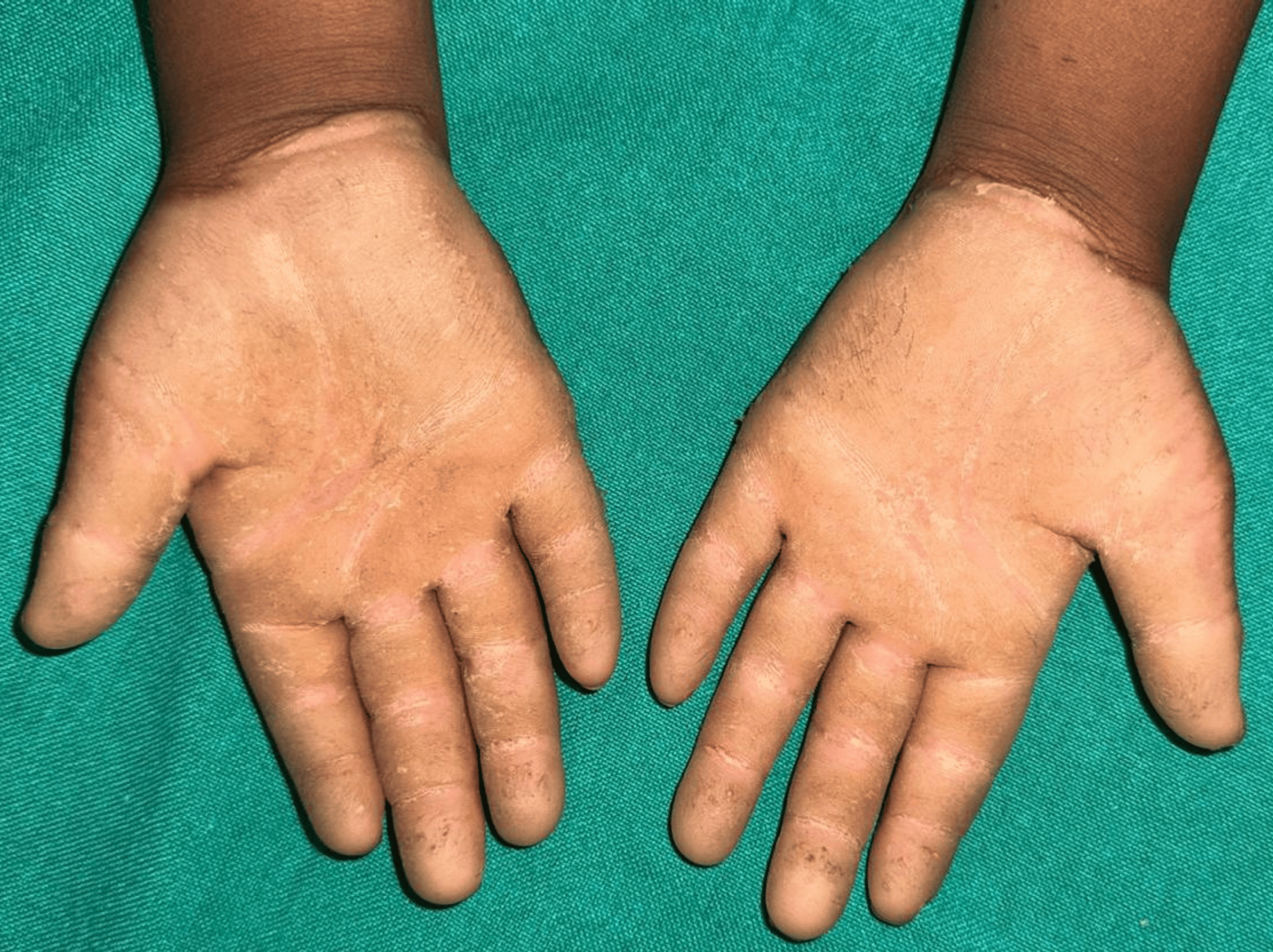
Diffuse, well-demarcated, yellow-orange hyperkeratotic plaques in the palms with fissures over pressure points

The palms of both hands were covered by diffuse, yellow-orange hyperkeratotic plaques, which had a well-demarcated margin at the transition to normal skin (Figure 1). The thickened skin measured approximately 3-4 mm in thickness, had a waxy texture, and showed few deep fissures (1-2 mm depth), particularly over pressure points such as the thenar eminence and along the palmar creases. Despite the pronounced keratosis, there was no surrounding erythema or inflammation.

**Figure 2:**
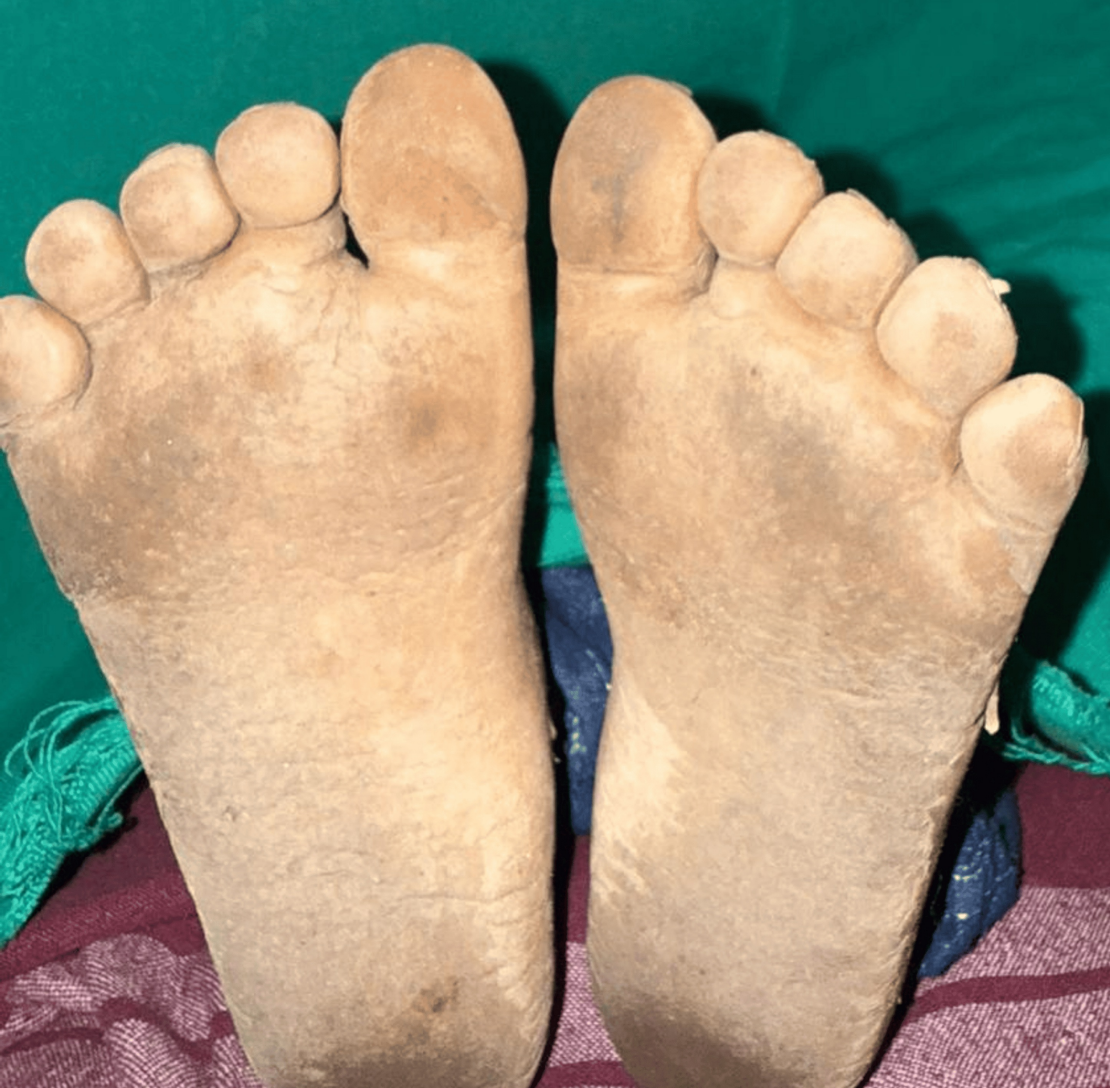
Diffuse plantar hyperkeratosis most pronounced on the heel, with multiple fissures

The soles of the feet also had diffuse hyperkeratosis (4-5 mm thick), with the thickening being most noticeable in areas that bear weight, such as the heels and the plantar aspect of the toes (Figure 2). The keratoderma on the soles had a yellow-brown hue and was associated with multiple painful fissures (2-3 mm depth), particularly in the heels. The thickened skin did not encompass the dorsal surfaces of the feet or extend beyond the ankles, suggesting a non-transgradient pattern.

Systemic examination

The child's fingers were flexible with a normal range of motion, and there were no contractures or constriction bands around the digits. The fingernails and toenails were normal in appearance (no nail dystrophy or clubbing). Hair on the scalp and body was of normal texture and density. There was no palmoplantar hyperhidrosis or malodor from the affected areas.

No cardiac murmurs, respiratory signs, organomegaly, or neurological deficits were detected. The child demonstrated a normal gait, though with evident discomfort when walking barefoot.

Laboratory investigations involving routine hematological and biochemical workups were uneventful (Tables 1-2). However, several key investigations warranted specific attention for diagnostic clarification and treatment planning.

**Table 1:** Serum biochemical profile assessing renal function, liver enzymes, and bilirubin fractions (07 February 2025) Values are presented alongside biological reference intervals appropriate for pediatric populations where applicable. BUN: blood urea nitrogen; SGOT: serum glutamic oxaloacetic transaminase; SGPT: serum glutamate pyruvate transaminase

Test Name	Result	Biological Reference Interval	Unit
Renal profile			
BUN	7	5–18	mg/dL
Creatinine	0.4	0.32–0.73	mg/dL
Sodium	131	136–145	mmol/L
Potassium	3.8	3.5–5.1	mmol/L
Chloride	96	98–107	mmol/L
Bicarbonate	22	22–29	mmol/L
Liver function test (LFT)			
SGOT	44	<40	IU/L
SGPT	22	<41	IU/L
Alkaline Phosphatase	127	142–335	IU/L
Total Protein	7	6.6–8.7	g/dL
Albumin	4.1	0–4 days: 2.8–4.4; 5 days–14 yrs: 3.8–5.4	g/dL
Globulin	2.9	2–3.5	g/dL
A:G Ratio	1.4	1.1–1.2	-
Bilirubin Profile			
Total Bilirubin	0.14	<1.2	mg/dL
Direct Bilirubin	0.07	<0.30	mg/dL
Indirect Bilirubin	0.07	0–1 day: 1.2–8.5; 1–2 days: 3.2–11.3; 3–5 days: 1.3–11.8; Adult: 0.1–1	mg/dL

**Table 2:** Clinical pathology – urine routine and microscopy (07/02/2025)

Test Name	Result	Biological Reference Interval
Colour	Straw Yellow/Clear	Straw Yellow/Clear
pH	6.0	4.8–7.4
Specific gravity	1.015	1.016–1.022
Glucose	Negative	Negative
Protein	Negative	Negative
Bilirubin	Negative	Negative
Ketone	Negative	Negative
Urobilinogen	Negative	Negative
Erythrocyte	2+	<5/HPF
Nitrite	Negative	Negative
Leukocyte	Negative	Negative
Pus cells	1–2	<5 Cells/HPF
RBC	1–2	0/HPF
Epithelial cells	NIL	NIL/HPF
Casts	NIL	NIL/HPF
Crystals	Not seen	Not seen

Clinically significant findings

The patient had hyponatremia and hypochloremia. However, both were not below a significant range, hence was corrected and reevaluated. Inflammatory markers were unremarkable (erythrocyte sedimentation rate (ESR): 12 mm/hr, C-reactive protein (CRP): 0.4 mg/dL), effectively ruling out underlying systemic inflammatory conditions that could present as secondary keratoderma (Table 3). This finding proved particularly valuable since chronic inflammatory states occasionally present with palmoplantar thickening as an early manifestation.

**Table 3:** Complete blood count (07/02/2025) ESR: erythrocyte sedimentation rate; LYMPH: lymphocytes; EOS: eosinophils; MONO: monocytes; BASO: basophils; PCV: packed cell volume; MCV: mean corpuscular volume; MCH: mean corpuscular hemoglobin; MCHC: mean corpuscular hemoglobin concentration

Test Name	Result	Biological Reference Interval
ESR 1 hr	12	3–13 mm/hr
Haemoglobin	10.5	11–14 gm/dL
TC	5870	5000–17,000 cells/cmm
POLY	43.8	30–40%
Immature granulocyte (Promyelo, myelo, metamyelo)	0.2	<1.0%
LYMPH	48.7	25–45%
EOS	1.7	1–6%
MONO	5.1	2–10%
BASO	0.5	0–1%
RBC count	5.01	4.5–5.2 mill/cmm
Platelet count	2.59	2.4–9 lakh/cumm
PCV	33.3	34–40%
MCV	66.5	75–87 fL
MCH	21.0	24–30 pg
MCHC	31.5	31–37 gm/dL
CRP	0.4	<10 mg/dL

Thyroid function studies demonstrated normal parameters (TSH: 2.1 μIU/mL), ruling out hypothyroidism - a recognized cause of secondary hyperkeratosis that can mimic inherited keratoderma patterns (Table 4). The normal thyroid status strengthened our clinical suspicion of primary sporadic keratoderma rather than metabolically driven skin changes.

**Table 4:** Lipid and thyroid profiles (08/02/2025) HDL: high-density lipoprotein; LDL: low-density lipoprotein; TSH: thyroid-stimulating hormone; FT4: free thyroxine

Test Name	Result	Reference Range	Unit
Total cholesterol	160	0–00	mg/dL
Triglyceride	78	0–150	mg/dL
HDL cholesterol	20	60–85	mg/dL
LDL	79	0–100	mg/dL
Cholesterol: HDL ratio	5.9	-	-
FT4	1.34	1.1–1.3	ng/dL
TSH	2.10	0.55–5.31	mIU/mL

Urinalysis showed no evidence of metabolic disorder, helping exclude rare metabolic disorders such as tyrosinemia type II, which characteristically presents with palmoplantar keratoderma alongside systemic manifestations. This negative finding supported the isolated nature of our patient's condition.

Pre-treatment assessment

Baseline liver function tests (SGOT: 28 U/L, SGPT: 30 U/L) and lipid profile (total cholesterol: 160 mg/dL, triglycerides: 78 mg/dL) established reference values essential for monitoring potential retinoid therapy complications. These parameters remained stable throughout the treatment period, confirming therapeutic safety.

Specialized evaluations

Multidisciplinary assessment revealed a noteworthy clinical finding that required careful evaluation. The initial electrocardiogram showed an inverted T wave in v1 lead, raising concerns about potential cardiac involvement that might suggest syndromic keratoderma variants. However, subsequent echocardiography demonstrated normal cardiac structure and function, effectively ruling out cardiocutaneous syndromes.

Comprehensive sensory organ evaluation through audiological and ophthalmological examinations yielded normal results. These findings proved crucial in excluding Vohwinkel syndrome and related disorders that combine palmoplantar keratoderma with sensory impairments.

The systematic exclusion of secondary causes, combined with negative family history and normal multisystem evaluation, strongly supported the diagnosis of isolated sporadic palmoplantar keratoderma. This comprehensive diagnostic approach ensured accurate classification while establishing safe parameters for targeted therapeutic intervention.

After three months of therapy, significant clinical improvement was observed. The thickness of the hyperkeratotic plaques diminished by approximately 50%, and there was a notable reduction in the depth and number of fissures. The patient reported minimal pain while walking, allowing for improved mobility and participation in physical activities at school. The palmar and plantar skin became more supple, with significantly reduced cracking under pressure or strain.

The patient continues to be under regular follow-up, with ongoing care focused on maintaining the improvement achieved with systemic therapy. The family was counseled on the chronic nature of the condition and the need for long-term adherence to skin care.

## Discussion

This case presents a sporadic form of diffuse non-transgradient palmoplantar keratoderma with onset age of two years, with no family history suggestive of hereditary transmission. The clinical presentation of diffuse, well-demarcated hyperkeratotic plaques with painful fissures and their presence particularly over pressure points is a characteristic feature of classic PPK. Both the absence of transgredient spread, i.e., extension beyond palmoplantar surfaces, and the mere lack of associated systemic findings after thorough evaluation stand in favor of the diagnosis of isolated PPK.

Previous literature has established various classification systems for PPK, with Unna-Thost, Vorner, and Greither types being among the well-described hereditary forms. Sporadic cases of PPK in children with early onset but with absence of family history are less frequently reported [4]. Yang et al., in their study, documented that some cases of PPK may apparently represent de novo mutations in genes associated with hereditary PPK, such as KRT1, KRT9, or SLURP1 [5]. Dev et al., in their study, explained about sporadic cases of PPK with clinical features resembling diffuse hereditary PPK but without identifiable genetic inheritance patterns [6], similar to our case.

The pathogenesis of sporadic PPK remains incompletely understood. While hereditary forms typically involve identified genetic mutations affecting keratinocyte differentiation or adhesion, sporadic cases may arise from de novo mutations or environmental factors influencing epidermal homeostasis. Recent research by Thomas et al. suggests that, even in the absence of family history, genetic analysis may reveal mutations in genes associated with hereditary PPK, implying that some sporadic cases represent new mutations rather than distinct pathophysiological entities [7].

A lot of conditions were ruled out in the diagnosis for us to arrive at sporadic diffuse PPK. Hereditary diffuse PPK (Unna-Thost or Vorner type) was considered, but it typically shows autosomal dominant inheritance and was ruled out. Acquired PPK secondary to inflammatory dermatoses (psoriasis, eczema) was ruled out by the absence of inflammation and other cutaneous findings. Acquired PPK secondary to systemic conditions (thyroid disorders, malignancy) was excluded through appropriate investigations. Syndromic PPK (Papillon-Lefèvre syndrome, Olmsted syndrome) was ruled out by the absence of associated features and mechanical hyperkeratosis - excluded based on the diffuse nature and early onset.

The systematic approach to diagnosis, including thorough history, physical examination, and targeted investigations, allowed for the establishment of sporadic PPK as the most likely diagnosis.

The decision to initiate systemic retinoid therapy was based on several factors, such as severity and chronicity of the condition (six-year duration), significant functional impairment affecting mobility, and the diffuse nature of involvement, which often responds poorly to topical therapies alone.

Acitretin, a second-generation retinoid, normalizes epidermal cell proliferation and differentiation through interaction with nuclear retinoic acid receptors. This mechanism directly addresses the pathophysiological basis of PPK involving abnormal keratinocyte proliferation and differentiation. The dose of 10 mg twice weekly was selected to balance efficacy with the risk of side effects in a pediatric patient.

Management of pediatric PPK presents several challenges. The chronic nature of the condition necessitates long-term treatment strategies that must balance efficacy with safety considerations, particularly for systemic therapies. Retinoids, while effective, require monitoring for potential adverse effects, including mucocutaneous dryness, hyperlipidemia, and hepatotoxicity. The six-weekly monitoring of lipid profiles and liver function in our patient was essential to ensure safe therapy.

Patient adherence represents another challenge, particularly for topical therapies that require consistent application. Engaging the family in treatment decisions and educating them about the chronic nature of PPK were crucial aspects of management.

## Conclusions

This case of sporadic diffuse PPK in a paediatric age group highlights the need for thorough clinical evaluation to differentiate isolated PPK from its syndromic variants. Response to systemic retinoid therapy (acitretin) indicates the effectiveness of this treatment in addressing severe, diffuse PPK, including instances lacking a family history. Constant follow-up throughout treatment facilitated safety and led to notable enhancements in symptoms and quality of life.

This case enhances the understanding of treatment outcomes in early-onset sporadic PPK and highlights the necessity of individualised therapy tailored to disease severity and functional impact. Long-term follow-up is crucial for monitoring disease recurrence and late-emerging systemic manifestations.
